# Efficient orbit-aware triad and quad census in directed and undirected graphs

**DOI:** 10.1007/s41109-017-0027-2

**Published:** 2017-06-15

**Authors:** Mark Ortmann, Ulrik Brandes

**Affiliations:** Department of Computer & Information Science, University of Konstanz, Box 67, Konstanz, 78457 Germany

**Keywords:** Graphlets, Motifs, Subgraph census, Graph statistics

## Abstract

The prevalence of select substructures is an indicator of network effects in applications such as social network analysis and systems biology. Moreover, subgraph statistics are pervasive in stochastic network models, and they need to be assessed repeatedly in MCMC sampling and estimation algorithms. We present a new approach to count all induced and non-induced four-node subgraphs (the quad census) on a per-node and per-edge basis, complete with a separation into their non-automorphic roles in these subgraphs. It is the first approach to do so in a unified manner, and is based on only a clique-listing subroutine. Computational experiments indicate that, despite its simplicity, the approach outperforms previous, less general approaches.

By way of the more presentable triad census, we additionally show how to extend the quad census to directed graphs. As a byproduct we obtain the asymptotically fastest triad census algorithm to date.

## Introduction

The $\mathcal F$-census of a graph is the frequency distribution of subgraphs from a family $\mathcal {F}$ of non-isomorphic graphs in an input graph. In this work we focus on four-node subgraphs, i.e. *quads*.

Discrimination of graphs by a subgraph census was proposed already by Holland and Leinhardt (1970; 1976) in the context of social networks and it is of utmost importance for the effects of exponential random graph models (Robins et al. 2007). While there is extensive work on determining the subgraph census for varying subgraph sizes (Kloks et al. 2000; Kowaluk et al. 2011; Lin et al. 2012) and also for directed graphs (Eppstein et al. 2010), the focus is almost always on the global distribution, i.e. the number of triangles a graph contains, but not on how often a given node is part of a triangle. For many properties characterizing nodes and edges it is however necessary to know the subgraph census on the node or edge level. For example, to calculate a node’s *clustering coefficient* we need to know in how many triangles it is contained. The same holds for the *Jaccard index* computed with respect to an edge. Although for these two examples it is not necessary to calculate the frequencies of all non-isomorphic induced 3-node subgraphs, i.e. the triad census, there exist edge weights that take different subgraph configurations into account (Auber et al. 2003) and the running time for most edge metrics (Melançon and Sallaberry 2008) is dominated by calculating the frequencies of particular subgraphs. Using the *k*-subgraph census on an edge level finds application in the context of extracting sparse graph representations that amplify group cohesion (Auber et al. 2003; Nick et al. 2013; Nocaj et al. 2015). While the approach by Nick et al. relies on the triad census, Nocaj et al. (2015) show that using a weighted quad census instead results in a superior sparsifier, as quads are more encompassing in reflecting local density. A further scenario where the quad census is of vital importance is in the evaluation of graph models with respect to the accuracy by which they resemble observed graphs (Pržulj et al. 2004).

While *k*-subgraph censuses specific for nodes and edges are not used widely in social network analysis, it is different for bioinformatics. So far, however, even here the use is restricted to connected *k*-node subgraphs, so called *graphlets* (Pržulj et al. 2004) or *motifs* (Milo et al. 2002).

**Fig. 1:**
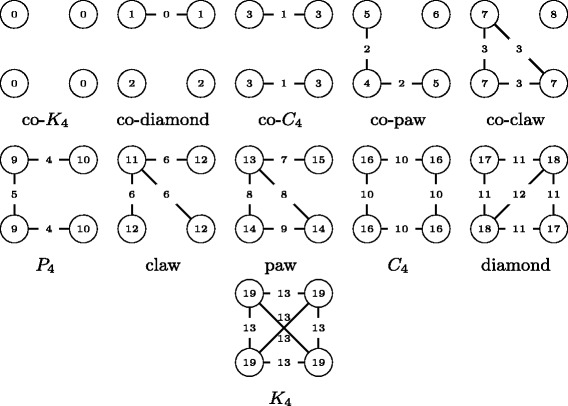
All non-isomorphic subgraphs with four nodes (quads). Node and edge labels refer to the orbits and were enumerated such that each orbit is identified with a single quad

A further differentiation of subgraph censuses consist in the distinction of node and edge automorphism classes (orbits) in each graphlet. For example, in a diamond (i.e. a complete four-node graph minus one edge), there are two node and edge orbits as shown in Fig. 1. The node orbits are defined by the nodes with degree 2 and 3, respectively. The edge orbits are determined by the edge connecting the nodes with degree 3 and all remaining edges, respectively. This differentiation by orbits is particularly interesting for the distinction of roles nodes and edges respectively fill in a quad. For example two nodes might have the same number of occurrences in a claw, cf. Fig. 1, which would lead to the assumption that they are similar, however by distinguishing the orbits we might see that the one node is always in orbit 11, and therefore in control of e.g. the information flow, while the other is always in orbit 12. That is the reason why the orbit-aware subgraph census has been used to mine central role structures in graphs (Doran 2014), but restricted to triads. Direct applications of the orbit-aware quad census can for example be found in the context of graph clustering (Milenković and Pržulj 2008; Solava et al. 2012).

Due to the importance of subgraph enumeration and censuses in bioinformatics, various computational methods (Hočevar and Demšar 2014; Marcus and Shavitt 2012; Milenković et al. 2008; Wernicke and Rasche 2006) were proposed.

The general approach to determine a subgraph census on the global level is to solve a system of equations that relates the non-induced subgraph frequency of each non-isomorphic *k*-node subgraph with the number of occurrences in other *k*-node subgraphs (Eppstein et al. 2010; Eppstein and Spiro 2009; Kloks et al. 2000; Kowaluk et al. 2011; Lin et al. 2012). It is known that the time needed to solve the system of equations for the four-node subgraph census, which we refer to as the *quad census*, on a global level is $\mathcal {O}(a(G)m + i(G))$ (Lin et al. 2012), where *i*(*G*) is the time needed to calculate the frequency of a given four-node induced subgraph in *G*, and *a*(*G*) is the *arboricity*, i.e. the minimum number of forests needed to cover *E*. Following the idea of relating non-induced and induced subgraph counts, Marcus and Shavitt (2012) present a system of equations for the orbit-aware connected quad census on a node level that runs in $\mathcal {O}(\Delta (G)m+m^{2})$ time with *Δ*(*G*) denoting the maximum degree of *G*. Because of the larger number of algorithms invoked by Marcus and Shavitt’s approach, Hočevar and Demšar (2014) present a different system of equations, again restricted to connected quads, that requires fewer counting algorithms and runs in $\mathcal {O}(\Delta (G)^{2}m)$ time, but does not determine the non-induced counts.

*Contribution:* We present the first algorithm to count both induced and non-induced occurrences of all four-node subgraphs (quads). It is based on a fast algorithm for listing a single quad type and capable of distinguishing the various roles (orbits) of nodes and edges. While this simplifies and generalizes previous approaches, our experimental evaluation indicates that it is also more efficient. Furthermore, we show using the example of the orbit-aware directed triad census a strategy to extend the orbit-aware quad census computation to directed graphs and thus obtain the asymptotically fastest algorithm for graph-level triad census computation along the way.

In the following section we provide basic notation followed by an introduction of the system of linear equations and the algorithm utilized in section “Determining the orbit-aware quad census”. In section “Runtime experiments” we present a running time comparison on observed and synthetic graphs showing that our approach is more efficient than related methods. Using the example of the triad census, we present in section “Triad census” a strategy to calculate the orbit-aware quad census of directed graphs without changing its asymptotic running time. We finally conclude in section “Conclusion”.

## Preliminaries

We consider finite simple undirected graphs *G*=(*V,E*) and denote the number of nodes by *n*=*n*(*G*)=|*V*| and the number of edges by *m*=*m*(*G*)=|*E*|. The *neighborhood* of a node *v*∈*V* is the set *N*(*v*)={*w* : {*v,w*}∈*E*} of all adjacent nodes, its cardinality *d*(*v*)=|*N*(*v*)| is called the *degree* of *v*, and *Δ*(*G*)= max*v*∈*V*{*d*(*v*)} denotes the maximum degree of *G*.

For finite simple directed graphs *G*=(*V,E*) we denote the *outgoing neighborhood* of a node *v*∈*V* by *N* ^+^(*v*)={*w* : (*v,w*)∈*E*}. The *incoming neighborhood* *N* ^−^(*v*) is defined analogously and we call *N* ^⇔^(*v*)=*N* ^+^(*v*)∩*N* ^−^(*v*) the mutual neighborhood. The underlying undirected graph *G* ^′^=(*V,E* ^′^) of a simple directed graph *G*=(*V,E*) has the edge set *E* ^′^={{*u,v*} : (*u,v*)∨(*v,u*)∈*E*}

A complete graph with *k* nodes is denoted by *K*_*k*_, and *K*_3_ is also called a *triangle*. We use $T(u)\,=\,{N(u)\choose 2} \cap E$ to refer to the set of node pairs completing a triangle with *u* and *T*({*u,v*})=*N*(*u*)∩*N*(*v*) for the set of nodes completing a triangle with the edge *e*={*u,v*}. For the cardinality of these sets we write *t*(*u*)=|*T*(*u*)| and *t*(*e*)=|*T*(*e*)|. A *triad* and a *quad* are any graphs on exactly three and four nodes.

A subgraph *G* ^′^=(*V* ^′^,*E* ^′^) of *G*=(*V,E*), *V* ^′^⊆*V*, is called (node-)*induced*, if $E' = {V' \choose 2} \cap E$, and it is called *non-induced*, if $E' \subseteq {V' \choose 2} \cap E$.

Two undirected graphs *G* and *G* ^′^ are said to be *isomorphic*, if and only if there exists a bijection *π*:*V*(*G*)→*V*(*G* ^′^) such that {*v,w*}∈*E*(*G*) ⇔ {*π*(*v*),*π*(*w*)}∈*E*(*G* ^′^). Each permutation, including identity, of the node set *V*, such that the resulting graph is isomorphic to *G* is called an *automorphism* and the groups formed by the set of automorphisms is denoted *automorphism class* or *orbit*.

## Determining the orbit-aware quad census

The *k*-node subgraph census is usually computed via a system of linear equations relating the non-induced and induced *k*-subgraph frequencies, as the non-induced frequencies are easier to compute. Lin et al. (2012) show that for *k*=4 all non-induced frequencies, except for *K*_4_, can be computed in $\mathcal {O}(a(G)m)$ time. This implies that the total running time to calculate the quad census at the level of the entire graph is in $\mathcal {O}(a(G)m+i(G))$, where *i*(*G*) is the time needed to compute the induced frequencies for some induced quad-type.

The approach of Lin et al. however, is not suitable to answer questions as to how often a node or an edge is contained in a *K*_4_. Furthermore, the automorphism class of the node/edge in the quad is sometimes of interest. All non-isomorphic graphs with four nodes are shown in Fig. 1 and the node/edge labels refer to their automorphism classes (orbits). For example in a diamond all edges of the *C*_4_ belong to the same orbit while the diagonal edge belongs to another. Analogously the orbits of the nodes can be distinguished.

As our approach also relies on relating non-induced and induced frequencies we will start by presenting how the non-induced frequencies for a node/edge in a given orbit relate to the induced counts. Thereafter, we will present equations to compute the respective non-induced frequencies and prove that our approach matches the running time of Lin et al., implying that it is asymptotically as fast as the fastest algorithm to compute the frequencies on a node and edge level for any induced quad. Note that in the following when we talk about non-induced frequencies we exclude those of the *K*_4_, as it equals the induced frequency.

### Relation of induced and non-induced frequencies

To establish the relation between induced and non-induced frequencies, the number of times *G* ^′^ is non-induced in any other graph *G* with the same number of nodes has to be known. For instance, let us assume that *G* ^′^ is a *P*_3_ and *G* a *K*_3_ (co-paw and -claw without isolated node cf. Fig. 1). Having the definition of the edge set for non-induced subgraphs in mind, we see that *G* contains three non-induced *P*_3_, as each edge can be removed from a *K*_3_ to create a *P*_3_. Consequently, if we know the total number of non-induced *P*_3_ and we subtract three times the number of *K*_3_ we obtain the number of induced *P*_3_ of the input graph.

Similarly, we can establish systems of equations relating induced and non-induced frequencies on a node and edge level distinguishing the orbits for quads, see Figs. 3 and 4.^1^ Note that both systems of equations are needed since we cannot compute the node from the edge frequencies and vice versa, but from both we can compute the global distribution. In the following we show the correctness for *ei*_10_(*e*).

**Fig. 2:**
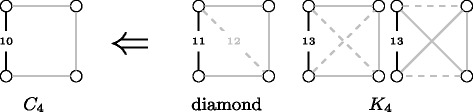
The three quads containing a non-induced *C*_4_. *Dashed lines* indicate that their removal creates a *C*_4_. Edge label correspond to orbits

*Induced orbit 10 edge census.* Let us assume we want to know how often edge *e* is in orbit 10 or in other words part of a *C*_4_. We know that a *C*_4_ is a non-induced subgraph of a diamond, *K*_4_ and of itself, cf. Fig. 2, and that there is no other quad containing a non-induced *C*_4_. Let us first concentrate on the diamond. In a diamond we have two different edge orbits; orbit 11, i.e. the edges on the *C*_4_, and orbit 12, i.e. the diagonal edge. Figure 2 shows that for every diamond where *e* is in orbit 12 there is no way to remove an edge, such that this graph becomes a *C*_4_, but for each diamond where *e* is in orbit 11 we can remove the diagonal edge and end up with a *C*_4_. Therefore, the non-induced number of subgraphs where *e* is in orbit 10 contains once the number of induced subgraphs where *e* is in orbit 11, but not those in orbit 12. As for the case of the *C*_4_ in a *K*_4_ all edges are in the same orbit. From a *K*_4_ we can construct a *C*_4_ containing a specific edge in two ways. The first is to remove both diagonal edges, cf. Fig. 2; and the second to delete the two horizontal edges. As a consequence the induced number of *e* being in orbit 10 is given by *ei*_10_(*e*)=*en*_10_(*e*)−*ei*_11_(*e*)−2*ei*_13_(*e*).

Following this concept all other equations can be derived.

### Calculating non-induced frequencies

**Fig. 3:**
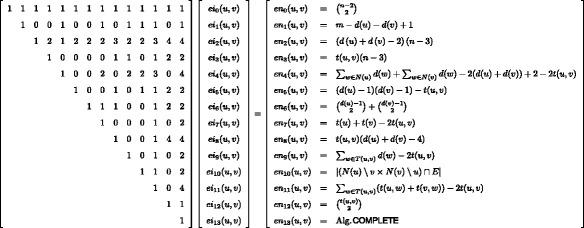
System of equations for orbit-aware quad census on an edge level. *ei* refers to induced and *en* to non-induced counts

**Fig. 4:**
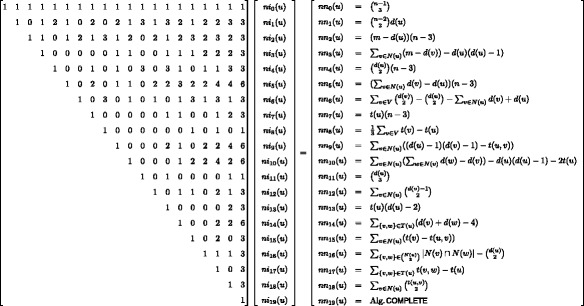
System of equations for orbit-aware quad census on a node level. *ni* refers to induced and *nn* to non-induced counts

The calculation of the non-induced frequencies is (computationally) easier than for the corresponding induced counts, except for *K*_4_s. This is due to the fact that the non-induced frequencies can be constructed from smaller, with respect to the number of nodes, subgraphs cf. Figs. 3 and 4. In the following we show the correctness of *nn*_14_(*u*) and *en*_4_(*u,v*).

*Non-induced orbit 14 node census.* To determine *nn*_14_(*u*) we start by enumerating all triangles containing *u*. Let *v* and *w* form a triangle together with *u*. As *u* is in orbit 14 we know that each neighbor of *v* and *w* that is not *u,v* or *w* definitely creates a non-induced paw with *u* in orbit 14; while this does not necessarily hold for neighbors of *u* as they might not be connected to *v* or *w* (and, if they are, we already gave credit to this). Note that even if a neighbor of either *v* or *w* is a neighbor of *u* as well there is no additional paw with *u* in orbit 14 and therefore $nn_{14}(u) = \sum _{\{v,w\}\in T(u)} (d(v)+d(w) -4)$.

*Non-induced orbit 4 edge census.* Edge *e*={*u,v*} is non-induced in orbit 4 for each *P*_3_ starting at *u* or *v* which neither contains *e* nor closes a *K*_3_ with *e*. The number of *P*_3_s starting at *u* not containing *e* equals $\sum _{w \in N(u) \setminus v}(d(w) - 1)$. However, the node *v* might be a neighbor of *w* and therefore there is a path of length two (via *w*) connecting *u* and *v*. Since this creates a three-node subgraph, more precisely a triangle, and not a quad we have to adjust for this by subtracting twice the number of triangles containing *e*. Consequently, $en_{4}(u,v) = \sum _{w \in N(u)} d(w) + \sum _{w \in N(v)} d(w) - 2(d(u)+d(v))+2 -2t(u,v)$.

In the following, we focus on the algorithm calculating all required frequencies to solve the systems of equations.

### Listing complete quads

In order to be able to solve the systems of equations we need to compute the non-induced quad counts as well as any of the induced frequencies. This requires an algorithm that is capable of solving the following tasks on a node and edge level:

1. Counting and listing all *K*_3_
2. Calculating non-induced *C*_4_ frequencies
3. Determine induced counts of any quad

We chose to calculate the induced counts for *K*_4_ to fulfill requirement 3. The reasons are a) to our knowledge there are no algorithms calculating induced counts on a node and edge level for any other quad more efficiently than the algorithm we are presenting here; b) a *K*_4_ has the property that all nodes and edges lie in the same orbit; c) all non-induced *C*_4_ can be counted during the execution of our algorithm. Since listing, also known as enumerating, all *K*_4_ has to solve the subproblem of listing all *K*_3_, we will start explaining our algorithm by presenting how *K*_3_s can be listed efficiently. Note that this algorithm satisfies requirement 1.

Listing all triangles in a graph is a well studied topic (Ortmann and Brandes 2014). We show in our previous work (Ortmann and Brandes 2014) that one of the oldest triangle listing algorithms, namely K3 by Chiba and Nishizeki (1985) is in practice the fastest. This algorithm is based on neighborhood intersection computations. To achieve the running time of $\mathcal {O}(a(G)m)$, Chiba and Nishizeki process the graph in a way such that for each intersection only the neighborhood of the smaller degree node has to be scanned. This is done by processing the nodes sequentially in decreasing order of their degree. The currently processed node marks all its neighbors and is removed from the graph. Then the number of marked neighbors of a marked node is calculated.


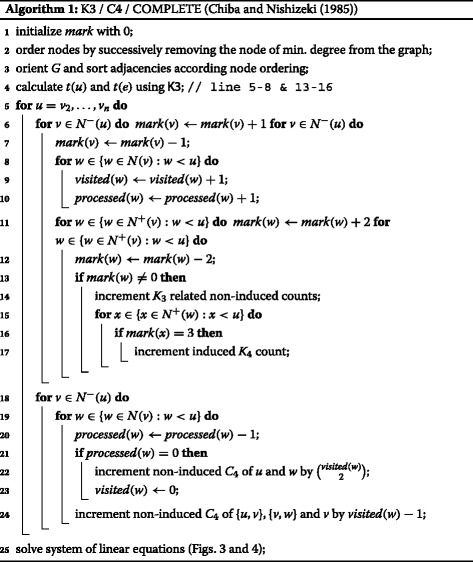


**Fig. 5:**
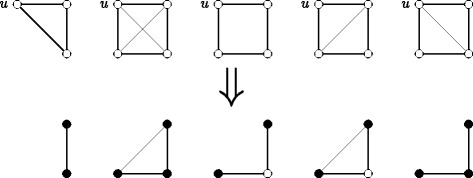
*Top*: Configurations that have to be found by our algorithm. *Bottom*: Resulting patterns to be detected when processing node *u*. Filled nodes are marked as neighbors of *u*

Let us think of this algorithm differently. When we process node *u* and remove it from the graph then every triangle that contains *u* is an edge where both endpoints are marked, cf. Fig. 5. This perception of the algorithm directly points us to a solution for the second and third requirement. As shown in Fig. 5, when node *u* is removed from the graph, every *K*_4_ that contains *u* becomes a *K*_3_ where all nodes are marked, implying that K3 can be easily adapted to list all *K*_4_s. Chiba and Nishizeki call this extension COMPLETE. Furthermore, only nodes that are connected to a neighbor of *u* can create a non-induced *C*_4_ and each *C*_4_ contains at least two marked nodes. Since all these nodes are processed already during the execution of algorithm K3 counting non-induced *C*_4_ on a node and edge level can be also done in $\mathcal {O}(a(G)m)$ time. The corresponding algorithm is called C4 in (Chiba and Nishizeki 1985) and the combination of these different algorithms is presented in Algorithm 1. It runs in $\mathcal {O}(a(G)^{2}m)$ (Chiba and Nishizeki 1985), and its novelty is that it follows the idea of directing the graph acyclic as we already proposed in the context of triangle listing (Ortmann and Brandes 2014). Furthermore, this acyclic orientation allows omitting node removals, and given the proper node ordering, it has the property that the maximum outdegree is bounded by $\mathcal {O}(a(G))$. Therefore, unlike for algorithm COMPLETE and C4 (Chiba and Nishizeki 1985), no amortized running time analysis is needed to prove that the running time is in $\mathcal {O}(a(G)^{2}m)$ and $\mathcal {O}(a(G)m)$, respectively, as we will show next.

***Runtime*** We will first show that the running time bound of our variant implementation of algorithm C4 is in $\mathcal {O}(a(G)m)$, therefore we ignore Lines 4, 6, 8, 12–19 and 27 of Algorithm 1 for now.

The running time of the remaining algorithm is given by the following equation:

$$\begin{array}{@{}rcl@{}} t(\textsf{C4}) & \leq & \sum\limits_{u \in V} d^{-}(u) + 2\sum\limits_{v \in N^{-}(u)}d^{-}(v) + d^{+}(v)\\ & = & m + 2 \sum\limits_{v \in V} d^{+}(v) (d^{-}(v)+d^{+}(v))\\ & \leq & m + 4m\Delta^{+}(G) \end{array} $$

As we order the nodes by successively removing the node of minimum degree from the graph, which can be computed in $\mathcal {O}(m)$ using a slightly modified version of the algorithm presented in (Batagelj and Zaveršnik 2003), it holds that *Δ* ^+^(*G*)<2*a*(*G*) (Zhou and Nishizeki 1994). The time required to initialize all marks is in $\mathcal {O}(n)$, orienting the graph is in $\mathcal {O}(n+m)$, and consequently the total running time is in $\mathcal {O}(a(G)m)$.

Let us now focus on the time required for calculating all *K*_4_s and therefore ignore Lines 9–11 and 20–27 of Algorithm 1 that is given by the following equation:

$$\begin{array}{@{}rcl@{}} t(\textsf{COMPLETE}) & \leq & \sum\limits_{u \in V} d^{-}(u) + \sum\limits_{v \in N^{-}(u)}2d^{+}(v) + \sum\limits_{w \in N^{+}(v)}d^{+}(w)\\ & \leq & m + \Delta^{+}(G)\sum\limits_{v \in V} 2d^{+}(v) + \sum\limits_{w \in N^{+}(v)}d^{+}(w)\\ & \leq & m + 2m\Delta^{+}(G) + \Delta^{+}(G) \sum\limits_{v \in V}d^{-}(v)\Delta^{+}(G)\\ & = & m + 2m\Delta^{+}(G) + m\Delta^{+}(G)^{2} \end{array} $$

By the same arguments it follows that our variant implementation of COMPLETE runs in $\mathcal {O}(a(G)^{2}m)$. Since Line 4 is in $\mathcal {O}(a(G)m)$ (Ortmann and Brandes 2014) and solving the systems of equations requires $\mathcal {O}(n+m)$ time, the overall complexity of Algorithm 1 is in $\mathcal {O}(a(G)^{2}m)$.

Before we give experimental evidence that our algorithm is not just asymptotically, but also in practice, superior to the currently fastest orbit-aware quad census algorithm, we want to give a more detailed explanation as to why algorithm C4 runs in $\mathcal {O}(a(G)m)$ instead of $\mathcal {O}(a(G)^{2}m)$, although every *K*_4_ contains three non-induced *C*_4_. The reason lies in the fact that COMPLETE belongs to the class of listing algorithms, while C4 is a counting algorithm. Since a listing algorithm has to enumerate every single occurrence of the subgraph of interest, its running time cannot be asymptotically faster than the number of subgraphs it has to list. For example every algorithm for listing all triangles in a graph cannot be asymptotically faster than *Θ*(*n* ^3^), since the complete graph contains ${n \choose 3}$ triangles. However, as counting does not require to enumerate every single triangle there exist algorithms with a lower worst-case complexity, e.g. via matrix multiplication (Coppersmith and Winograd 1990). This difference and the fact that in the non-induced scenario we can ignore the existence of some edges, explain the asymptotical differences between the two algorithms.

## Runtime experiments

We provide experimental evidence that our approach is not only asymptotically faster but also more efficient in practice than the currently fastest orbit-aware quad census algorithm. Comparison is restricted to the *orca software (v1.0)* implementing the approach of Hočevar and Demšar (2014), as the authors show that it is superior to other software tools in the context of quad census computation. Additionally, it is the only software we are aware of which can compute the orbit-aware quad census on an edge level, even if only for connected quads. To the best of our knowledge, except in the orca code, there is no other documentation of their approach.

### Setup and data

We implemented our approach in C++ using the *Standard Template Library* and compiled the code with the g++ compiler version 4.9.1 set to the highest optimization level. The *orca software* is freely available as an R package. To avoid measuring errors due to the R and C++ interface communication we extracted the C++ code and cleaned it from all R dependencies.

The tests were carried out on a single 64-bit machine with an 3.60GHz quad-core Intel Core i7-4790 CPU, 32GB RAM, running Ubuntu 14.10. The times were measured via the gettimeofday command with a resolution up to 10^−6^ seconds. We ran the executable in a single thread and forced it to one single core, which was dedicated only to this process. Times were averaged over 5 repetitions.

***Data*** We compared both approaches on a number of real world networks. The *Facebook100 dataset* (Traud et al. 2011) comprises 100 Facebook friendship networks of higher educational institutes in the US with network sizes of 762≤*n*<41*K* nodes and 16*K*<*m*<1.6*M* edges. Although these networks are rather sparse, they feature a small diameter, thereby implying a high concentration of connected quads. Apart from this we tested the algorithms on a variety of networks from the *Stanford Large Network Set Collection* (Leskovec and Krevl 2014). The downloaded data were taken from different areas to have realistic examples that encompass diverse network structures.

Additionally, we generated synthetic networks from two different models. The one class of generated graphs are small-worlds, which were created by arranging nodes on a ring, connecting each one with its *r* nearest neighbors, and then switching each dyad with probability *p*. The other class of graphs was drawn from a preferential attachment like model. Here we added *n* nodes over time to the initially empty network and each new node *v* connects to *r* existing nodes, each of which either chosen by preferential attachment or with probability *p* randomly from $\bigcup _{u \in N(v)}N(u)$. We generated graphs with fixed *n*=20000 and varying average degree as well as graphs with *n*∈{50000,140000,…,500000} and gradually increasing average degree. Four graphs were generated for each parameter combination.

We refer the reader to (Ortmann and Brandes 2014) for a more detailed description of the utilized graph models, the tested Stanford graphs, the chosen average degree, and parameters *r* and *p*.

### Results

**Fig. 6:**
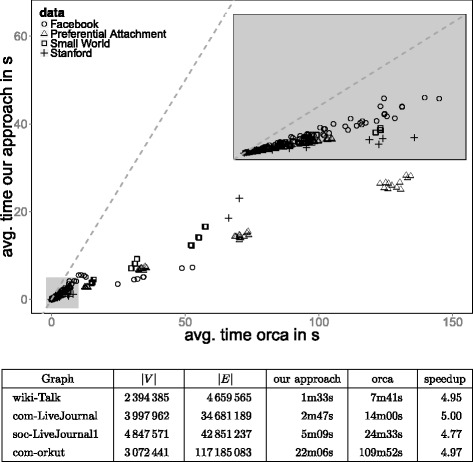
*Top*: Avg. running time of *orca* vs. avg. running time of our approach in seconds for all but the largest SNAP graphs. *Dots* below the main diagonal indicate that the algorithm on the y-axis is faster. Embedded plot displays *gray* area in higher resolution. *Bottom*: Time comparison for the largest SNAP graphs

In Fig. 6 we present the results of our experiments. In the top subfigure we plotted the avg. running time of *orca* against the avg. time needed by our approach for all but the largest Standford graphs. Each point that lies below the main diagonal indicates that our approach is faster. Consequently, the picture makes it clear that our algorithm is faster than the *orca software* for each tested network, even though we compute the whole node and edge orbit-aware quad census. The same findings are obtained for the larger graphs taken from SNAP.

The speed-up we achieve lies between 1.6 and 10 for the tested graphs. In general, however, the speed-up should be in *Θ*(log*Δ*(*G*)) for larger graphs. The reason is that, once *n* exceeds 30*K*, the algorithm implemented in the orca software runs in $\mathcal {O}(\Delta (G)^{2}m\log \Delta (G))$, instead of $\mathcal {O}(\Delta (G)^{2}m)$. The logarithmic factor originates from the time required for adjacency testing. While the orca software uses an adjacency matrix for these queries for graphs with *n*≤30*K*, it takes log*Δ*(*G*) for larger graphs (binary search), since no adjacency matrix is constructed. In contrast Algorithm 1 requires only $\mathcal {O}(n)$ additional space to perform adjacency tests in constant time. Note that orca’s algorithm using the adjacency matrix appears to follow the ideas of Chiba and Nishizeki, yet without exploiting the potential of utilizing a proper node ordering. Besides the faster *K*_4_ algorithm, another important aspect explaining the at least constant speed-up of our approach is our system of equations. For both the node and edge orbit-aware quad census Hočevar and Demšar do not calculate the exact non-induced counts. This requires that each induced subgraph with 3 nodes is listed several times and, more importantly, also non-cliques, which is not the case in our approach.

## Triad census

So far we have shown a general framework building on relating non-induced and induced frequencies to compute the orbit-aware *k*-subgraph census on a node and edge level basis using the example of quads. While this approach was restricted to simple undirected graphs, we show in the following how it can be extended to directed graphs. However, since the number of non-isomorphic directed quads is already 218 (Davis 1953), we will introduce this framework in the context of the (directed) triad census. As the required modifications for node and edge orbit-awareness are the same we will restrict our explanations to the node orbit-aware triad census computation. Note that since solving the (directed) quad census relies on non-induced frequencies of smaller, i.e. subgraph of size less than four, the distinctions of directed triads is required in order to solve the quad census for directed graphs and therefore some of the following equations are necessary for its computation.

**Fig. 7:**
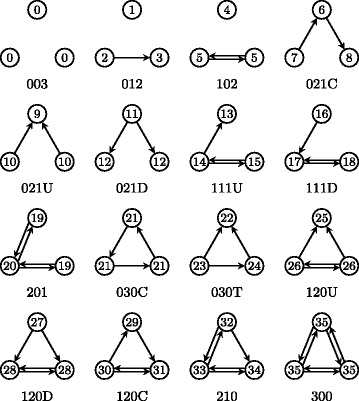
All directed non-isomorphic subgraphs with three nodes (triads). The graph labeling is based on the number of mutual, asymmetric, and null dyads in each triad with an additional indicator where needed (Wasserman and Faust 1994). Node labels refer to the orbits and were enumerated such that each orbit is identified with a single triad

The triad census of a graph denotes the frequency distribution of all non-isomorphic directed triads, cf. Fig. 7, in an input graph and finds application, among others, in social sciences e.g. to compare different graphs (Faust 2007; Wasserman and Faust 1994) or to extract distinct roles in networks (Doran 2014). The probably first algorithm to compute the triad census on a graph level is attributed to Moody (1998) with a running time of $\mathcal {O}\left (n^{2.376}\right)$ (Coppersmith and Winograd 1990). While this approach relies on matrix multiplication, Batagelj and Mrvar (2001) propose a combinatorial algorithm calculating the triad census in $\mathcal {O}(\Delta (G)m)$ time. Like Batagelj and Mrvar’s approach the proposed technique by Eppstein et al. (2010) requires to enumerate all connected triads. However, using a proper data structure allows them to further reduce the asymptotical complexity to an amortized $\mathcal {O}(h(G)m)$ running time where *h*(*G*) is the largest integer such that there exist *h* nodes of degree at least *h* (Hirsch 2005). Yet still the algorithm of Eppstein et al. is not optimal, as we will show in the following, since, as it is the case for the quad census, it is sufficient to list only all complete triads, which is asymptotically faster.

**Fig. 8:**
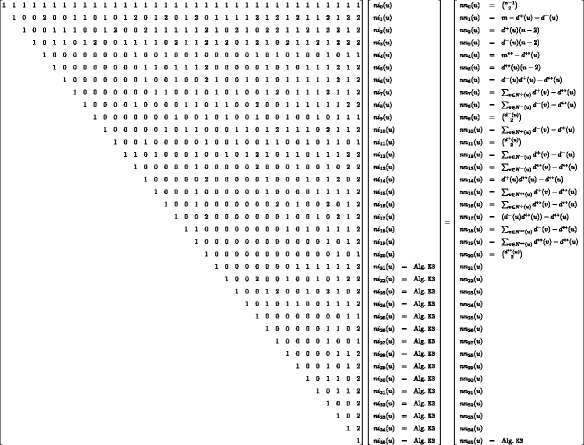
System of equations for orbit-aware directed triad census on a node level. *ni* refers to induced and *nn* to non-induced counts


Following the framework presented in the context of the undirected quad census we can relate orbit-aware non-induced and induced triad census frequencies via a system of linear equations as presented in Fig. 8. Since deriving this system of linear equations follows exactly the same strategy we presented earlier we omit the correctness proofs here. Although the system of linear equations in Fig. 8 requires the computation of several induced frequencies, compared to only one in the undirected case, cf. Figs. 3 and 4, we can make the following observation. All the induced orbit frequencies, i.e. 21 to 35, are triangles in the underlying undirected graph. Since each triangle in the underlying undirected representation *G* ^′^ of *G* corresponds to a directed triangle in *G*, and vice versa, we can list all triangles, *T*(*G* ^′^), in *G* ^′^ and then calculate the orbits of the nodes in each triangle *t*∈*T*(*G* ^′^) w.r.t. *G*. This directly implies that, since orbit 0 to 20 can be computed in $\mathcal {O}(m)$ which matches the running time to construct *G* ^′^, that the total running time of the orbit-aware triad census on a node level is in $\mathcal {O}(a(G)m + \sum _{t \in T(G)}o(t))$, since *m*(*G* ^′^)≤*m*(*G*). The $\mathcal {O}(a(G)m)$ factor is the running time of Chiba and Nishizeki’s algorithm K3 to list all triangles in a graph (Chiba and Nishizeki 1985; Ortmann and Brandes 2014), and *o*(*t*) denotes the complexity to compute the orbit of each node in a triangle. In the following we will show that $o(t) \in \mathcal {O}(1)$ and therefore the time complexity for the computation of the orbit-aware triad census on a node level in $\mathcal {O}(a(G)m)$. As the (orbit-aware) triad census on a graph level can be computed from the node level, and since *a*(*G*)≤*h*(*G*) (Lin et al. 2012), this implies that our approach is not just easier to implement than the currently best algorithm for the triad census computation (Eppstein et al. 2010) on a graph level, but also asymptotically faster.

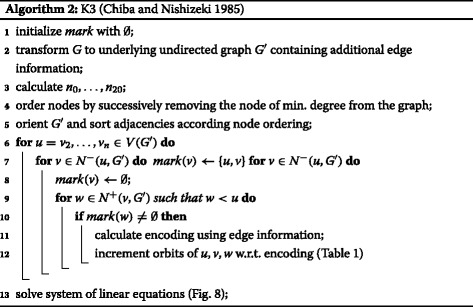


The idea of working on *G* ^′^ rather than on *G* for the computation of the triad census has already been used by Batagelj and Mrvar (2001). In order to relate the undirected triad *u*,*v,w*∈*V* in *G* ^′^ with its directed version in *G* they propose to map a triad to a number computed by the following formula

$$\text{code}(u,v,w) = l(u,v) + 2l(u,w) + 4l(v,u) + 8l(v,w)+16l(w,u) + 32l(w,v) $$

with *l*(*i,j*)=1 if (*i,j*)∈*E*(*G*) and 0 otherwise. Since this mapping is unique for each possible triad each number encodes exactly one of the 16 non-isomorphic triads, cf. Fig. 7. Furthermore, as we know for each possible triad the orbits of the nodes, we can extend this mapping to also encode the node orbits, cf. Table 1. Note that Table 1 contains all codes, yet our approach requires only those entries encoding orbits larger than 20. Since Table 1 allows us in constant time to map the code of *u*,*v,w* to their orbits, it remains to show that the computation of the encoding can also be done in constant time and therefore $o(t) \in \mathcal {O}(1)$.

**Table 1:** Mapping of code(*u,v,w*) to the triad and the orbits of *u,v,w*

Code	Triad	Orbits			Code	Triad	Orbits			Code	Triad	Orbits			Code	Triad	Orbits		
		*u*	*v*	*w*			*u*	*v*	*w*			*u*	*v*	*w*			*u*	*v*	*w*
0	003	0	0	0	16	012	3	1	2	32	012	1	3	2	48	021D	12	12	11
1	012	2	3	1	17	021C	6	8	7	33	021U	10	9	10	49	030T	24	22	23
2	012	2	1	3	18	102	5	4	5	34	021C	7	8	6	50	111U	15	13	14
3	021D	11	12	12	19	111U	14	13	15	35	030T	23	22	24	51	120U	26	25	26
4	012	3	2	1	20	021U	9	10	10	36	021C	8	6	7	52	030T	22	24	23
5	102	5	5	4	21	111D	17	18	16	37	111D	18	17	16	53	120D	28	28	27
6	021C	6	7	8	22	111D	17	16	18	38	030C	21	21	21	54	120C	31	29	30
7	111U	14	15	13	23	201	20	19	19	39	120C	30	31	29	55	210	33	34	32
8	012	1	2	3	24	021C	8	7	6	40	102	4	5	5	56	111U	13	15	14
9	021C	7	6	8	25	030C	21	21	21	41	111D	16	17	18	57	120C	29	31	30
10	021U	10	10	9	26	111D	18	16	17	42	111D	16	18	17	58	201	19	19	20
11	030T	23	24	22	27	120C	30	29	31	43	120D	27	28	28	59	210	32	34	33
12	021D	12	11	12	28	030T	22	23	24	44	111U	13	14	15	60	120U	25	26	26
13	111U	15	14	13	29	120C	31	30	29	45	201	19	20	19	61	210	34	33	32
14	030T	24	23	22	30	120D	28	27	28	46	120C	29	30	31	62	210	34	32	33
15	120U	26	26	25	31	210	33	32	34	47	210	32	33	34	63	300	35	35	35

With minor modifications it is possible to enable algorithm K3 to list, besides all nodes, also all edges belonging to a triangle in *G* ^′^, while not changing the algorithms asymptotic running time. If we further attach during the transformation from *G* to *G* ^′^ to each edge the information how it is directed in *G*, we can access *l*(*i,j*) in constant time. Consequently, we can compute code (*u,v,w*) and therefore *o*(*t*) in $\mathcal {O}(1)$. Note that if *N* ^⇔^ and *d* ^⇔^(*u*) are not part of the input they can also be computed during the construction of *G* ^′^. Even though the described algorithm can be directly derived from Algorithm 1, for convenience we present in Algorithm 2 the orbit-aware triad census on a node level algorithm. Since the additional work that has to be done compared to plain triangle listing is in $\mathcal {O}(m)$, we refer the reader to the evaluation of triangle listing algorithms in (Ortmann and Brandes 2014) to get an impression of the practical running times. Note that the presented strategy can also be used for orbit-awareness on an edge level without changing the asymptotic running time and that it can be directly applied to derive the orbit-aware directed quad census.

## Conclusion

We presented two systems of equations that enable us to efficiently determine the orbit-aware quad census of a graph down to the level of nodes and edges by applying an efficient single-subgraph listing algorithm and its subroutine. It was shown how induced and non-induced frequencies relate to one another and that we can compute the non-induced frequencies in $\mathcal {O}(a(G)m)$ time. This matches the best known running time bound for the more restricted non-induced quad census on the graph level, i.e. oblivious to the specific nodes and edges involved in each quad. With Algorithm 1 we showed a routine that is capable of computing all non-induced frequencies and listing all *K*_4_ while running in $\mathcal {O}(a(G)^{2}m)$ time, which is the asymptotically best known running time bound for listing any induced quad. This implies that the total running time of our approach matches the best known running time for quad census computation on a graph level in sparse graphs (Lin et al. 2012). In experiments we were able to show that the simplicity of our system of equations in combination with this efficient algorithm outperforms the currently best software to calculate the quad census.

Furthermore, using the example of the orbit-aware directed triad census on the node level, we outlined a strategy to extend the orbit-aware quad census on both the node and edge level to directed graphs. As a byproduct, we presented with Algorithm 2 the asymptotically fastest algorithm for the triad census computation on the graph level. We note that both algorithms can be parallelized with little effort.

## Endnote

^1^ Note that the preliminary version contained several typing errors.
